# Erucic acid exposure during the first year of life—Scenarios with precise food‐based dietary guidelines

**DOI:** 10.1002/fsn3.2652

**Published:** 2021-11-07

**Authors:** Mathilde Kersting, Hermann Kalhoff, Bernd Honermeier, Kathrin Sinningen, Thomas Lücke

**Affiliations:** ^1^ Research Department of Child Nutrition University Hospital of Pediatrics and Adolescent Medicine St. Josef‐Hospital Ruhr‐University Bochum Bochum Germany; ^2^ Pediatric Clinic Dortmund Dortmund Germany; ^3^ Institute of Agronomy and Plant Breeding I Justus Liebig University Giessen Germany

**Keywords:** exposure scenarios, food contaminants, infant nutrition, rapeseed oil, total daily diet

## Abstract

Recently, the European Food Safety Authority (EFSA) issued a tolerable daily intake (TDI) for erucic acid, which is mainly found in rapeseed oil. Infants may be exposed to erucic acid from rapeseed oil indirectly through maternal consumption via breastmilk or the fat component in formula, and directly as a part of complementary feeding (CF). To check the safety of infant nutrition, scenarios for erucic acid exposure were calculated based on the daily food amounts of the German dietary guidelines. Information on erucic acid concentrations in foods was obtained from European studies for breastmilk, from EFSA samples for formula powder, and from a representative analysis of rapeseed oil samples in the German retail market. 6 scenarios were calculated for the early milk feeding phase (4 formula feeding, 2 breastfeeding) and 8 scenarios for the later CF phase (5 CF +formula feeding, 3 CF +breastfeeding). Out of the 14 scenarios, only 3 resulted in exposures that were definitively below the TDI (range 4.4.–6.0 mg/kg bodyweight; BW). Assuming either high consumption or high concentration led to high exceedances (range 7.5–26.2 mg/kg BW), especially in case of the new EU limits for formula or vegetable oils (33.6 and 43.2 mg/kg BW, respectively). In our scenarios, high erucic acid exposures occurred during a particularly sensitive developmental period. To definitively weigh the potential risks from erucic acid in infants against nutritional benefits of the dietary recommendations, reliable, timely data on erucic acid in breast milk and formula are needed, similar to those from rapeseed oil in Germany.

## Graphical abstract

In infancy, the diet requires both, an especially high nutritional quality and toxicological safety. In view of the recent TDI for erucic acid issued by EFSA, our scenarios show that the nutritionally safe food‐based dietary guidelines for infancy in Germany may no longer be safe with regard to potential exposure to erucic acid that originates mainly from rapeseed oil. In addition to the strong data base on erucic acid in rapeseed oil in Germany, timely data bases for human milk and formula are needed to definitely decide on the toxicological safety of infant nutrition.

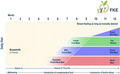

## 1 INTRODUCTION

In the first year of life, nutrition changes more than in any other period of later life, from exclusive breastfeeding in the first months to complementary feeding and to the family diet (ESPGHAN, 2017). The high nutritional requirements (per kg bodyweight, BW) and the still‐developing body functions and organ systems make infants especially susceptible to potential risks from hazardous substances in the diet. This is one of the reasons why foods for infants and young children are specifically regulated in European food law (EFSA, 2014; European Commission, 2016).

A special feature of infant nutrition is the relatively high‐fat intake to meet the high energy requirements for growth. In addition, dietary fat sources must ensure a balanced supply of fatty acids. While breast milk as the "gold standard" fully covers the high‐fat requirement in early infancy regardless of national boundaries and traditions, complementary foods already reflect typical dietary habits within countries. In the German food‐based dietary guidelines for infant nutrition, namely the “Dietary Scheme for the first year of life,” rapeseed oil has been recommended since many years as component of complementary feeding, not only because of the quantity of fat but also because of its balanced fatty acid pattern. To recommend rapeseed oil for human nutrition is possible nowadays, since rapeseed oil varieties with low levels of erucic acid, a monounsaturated omega‐9 fatty acid, with unfavorable sensory properties and suspected toxicological risks are available.

The level of erucic acid in foods varies widely among the most important *Brassicaceae* food sources, depending on the species and cultivar. Erucic acid content may account for more than 40% of the total fatty acids in natural forms of rapeseed and mustard seed, but only 0.5% in commercially bred cultivars of rapeseed developed since the 1970s (EFSA, 2016). The EU food regulation established maximum concentrations for erucic acid for vegetable fats and oils and foods containing these oils, as well as for infant and follow‐up formula.

Upon request of the European Commission, the European Food Safety Authority (EFSA) recently released for the first time a Tolerable Daily Intake (TDI) of erucic acid (7 mg/kg BW and day), resulting from a toxicological assessment based on animal models and common safety considerations (EFSA, 2016). Among all food groups, by far, the highest concentrations of erucic acid were found in rapeseed oil. In EFSA's assessments of erucic acid exposure across age groups, infants and young children were the only age groups that showed (slight) exceedances of the TDI. As an immediate consequence, the maximum levels of erucic acid allowed in the European Union in vegetable oils and in infant and follow‐on formula have been substantially reduced (European Commission, 2019a; b).

### 1.1 Objective

Against this toxicological background, the safety of the German food‐based dietary guidelines for infant nutrition has to be checked, as rapeseed oil is explicitly recommended there.

## 2 METHODS

### 2.1 Starting point

A main objective of food‐based dietary guidelines was to put nutrient reference values into practice considering existing dietary habits within the population (Clay, 1998). In infant nutrition, additionally, sensory‐motor development has to be considered. Thus, the German “Dietary Scheme” for the first year of life shows 3 phases of dietary development with smooth transitions between them (Kersting et al., 2020): exclusive breastfeeding during the first months, introduction of complementary feeding along with partial breastfeeding during the following second 6 months of life, and introduction of family diet toward the end of the first year of life (EFSA, 2019). Infant formula is used as a breastmilk substitute in the first months of life and follow‐on formula in the complementary feeding phase.

**FIGURE 1:**
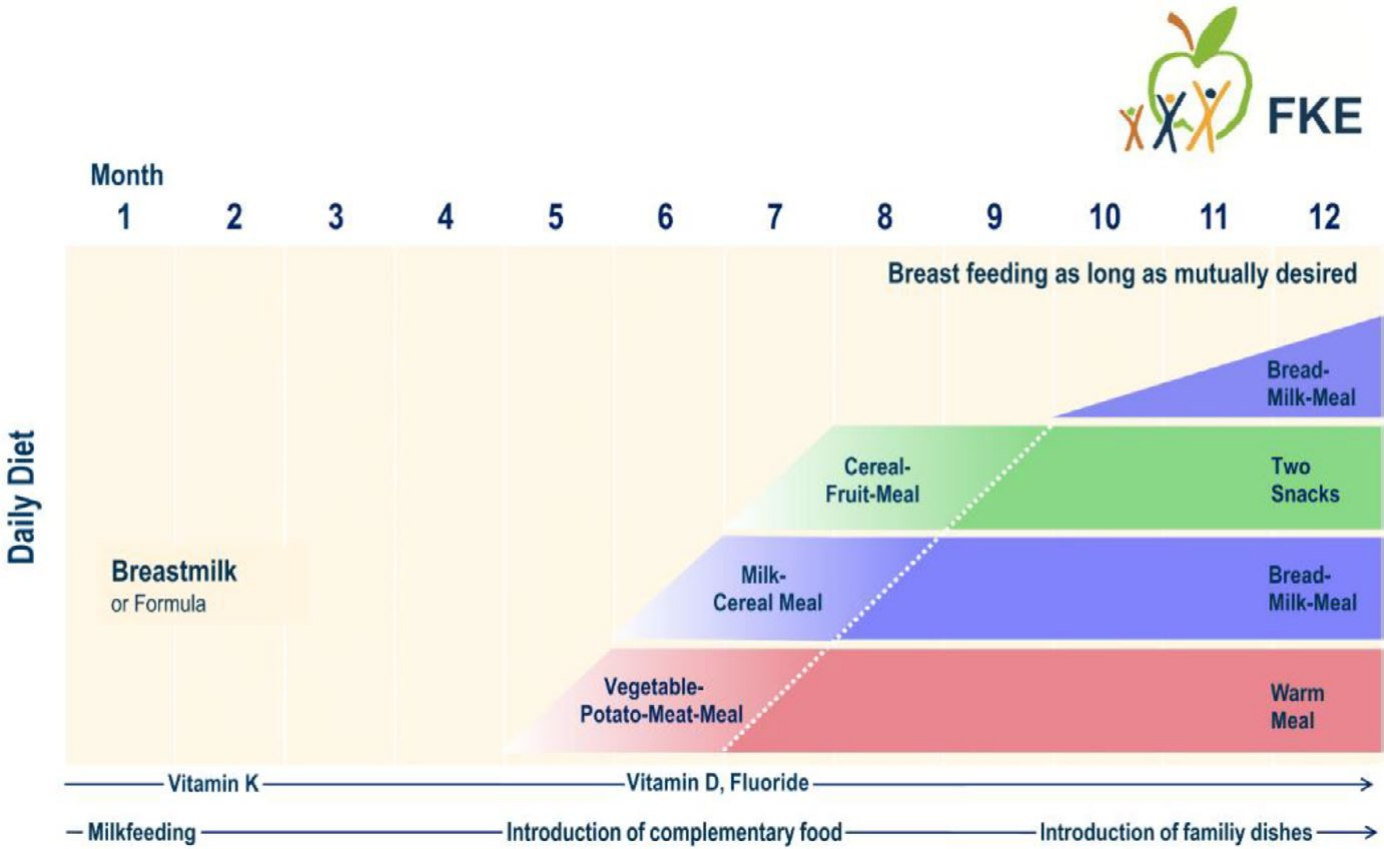
Dietary scheme for the first year of life

Here, for the exposure calculations, the age of approximately 1 month with the highest consumption per kg BW (due to the highest energy requirements for growth) was chosen for the first phase of exclusive milk feeding. The age of approximately 8 months was chosen for the second phase of complementary feeding, when the 3 daily complementary meals of the Dietary Scheme have been fully introduced (Figure 1). Balanced nutrient intake with the Dietary Scheme, measured against current European (EFSA) reference values, has recently been demonstrated (Kersting et al., 2020).

The exposure scenarios for the 2 phases followed the same calculation principle:

Erucic acid exposure =food consumption volume per kg BW per day x erucic acid concentration in food (from different data sources, as available).

### 2.2 Scenarios with exclusive milk feeding (first phase)

#### 2.2.1 A1 formula feeding

In a special guidance document on the risk assessment of substances present in food for infants, EFSA has recently derived data for infant formula consumption based on measured intakes during the first 16 weeks of life (EFSA, 2017). Maximum consumption volumes/kg BW were determined at about 1 month of age. Here, the consumption levels p50 (199 g/kg BW) and p95 (255 g/kg BW) for boys were used.

Data on erucic acid concentration in commercial formula as marketed were derived from an EFSA data collection of food samples, including infant formula powder, from European countries between 2005 and 2015 (218 samples, middle bound [MB] concentration: 253 mg/kg powder) (EFSA, 2016). For the conversion of infant formula powder to liquid milk, a factor of 1:8 (12% powder) was applied following EFSA (EFSA, 2017).

For the scenario with the current EU maximum level of erucic acid in formula (0.4% of total fat content (European Commission, 2019a), translation into liquid milk was enabled by using the upper permitted contents of fat (6.0 g/100 kcal) and energy (70 kcal/100 mL) of infant and follow‐on formula in the European Union (European Commission, 2016).

A1: Formula feeding, 4 scenarios were calculated:

1. Consumption p50; concentration (MB) of European samples of infant formula (EFSA)
2. Consumption p95; concentration (MB) of European samples of infant formula (EFSA)
3. Consumption p50; concentration maximum permitted EU level of formula (EU)
4. Consumption p95; concentration maximum permitted EU level of formula (EU)

#### 2.2.2 A2 breastfeeding

Consumption data for exclusive breastfeeding point to slightly lower consumption levels (10%–15%) than for formula (Dewey, 1998). For estimating the peak consumption of breastmilk (per kg BW), the formula consumption of scenario A1 was pragmatically reduced accordingly. Erucic acid concentration in mature human milk was estimated based on an updated EFSA review (EFSA, 2016), as an average value from 5 studies in European countries (Beijers & Schaafsma, 1996; Mihalyi et al., 2015; Szlagatys et al., 2013; Thakkar et al., 2019; Yuhas et al., 2006,).

A2. Breastfeeding, 2 scenarios were calculated:

1. Consumption p50 (formula p50—10%); concentration European studies
2. Consumption p95 (formula p95—10%); concentration European studies

### 2.3 Scenarios complementary feeding +milk (second phase)

#### 2.3.1 Data sources

Recipes of the 3 daily complementary meals of the Dietary Scheme at about 8 months of age are shown in Table 1.

**TABLE 1:** Dietary Scheme for the first year of life—Recipes of the three daily complementary meals at the age of about 8 months

First meal		Second meal		Third meal	
Home prepared					
Vegetable–potato‐–meat–meal		Milk–cereal–meal		Cereal–fruit–meal	
100 g	Vegetables	200 g	Milk	20 g	Cereals
50 g	Potatoes	20 g	Cereals	90 g	Water
30 g	Meat/Fish	20 g	Juice/Fruit	100 g	Fruit
5 g	Rapeseed oil			5 g	Rapeseed oil

Per day, 10 g rapeseed oil is added, 5 g in the vegetable‐potato‐meat meal, and 5 g in the cereal‐fruit meal because the ingredients of these meals are low in fat (Kersting et al., 2020).

For the erucic acid concentration in rapeseed oil, the P50 and P95 values of a recent representative survey of rapeseed oil samples in the German food retail market were used (Russo et al., 2021). In additional scenarios, the new reduced maximum erucic acid concentration in vegetable oils in the EU (20 g/kg) was assumed (European Commission, 2019b).

For the remaining milk part in the daily diet (200 mL), 3 assumptions were applied:

- Breastmilk (5 European studies)
- Follow‐on formula (EFSA data collection: 191 samples, concentration (MB): 187 mg/kg powder) (EFSA, 2016)
- Maximum permitted EU value for formula (European Commission, 2019b)

B1: Complementary meals +follow‐on formula; 5 scenarios.

1. Rapeseed oil (10 g); p50 analysis Germany +formula (200 mL) European samples (EFSA)
2. Rapeseed oil (10 g); p50 analysis Germany +formula (200 mL) max. perm. level (EU)
3. Rapeseed oil (10 g); p95 analysis Germany +formula (200 mL) European samples (EFSA)
4. Rapeseed oil (10 g); p95 analysis Germany +formula (200 mL) max. perm. level (EU)
5. Rapeseed oil (10 g); max. proposed level EU +formula (200 mL) max. perm. level EU

B2: Complementary meals +breastmilk; 3 scenarios.

1. Rapeseed oil (10 g); p50 analysis Germany +breastmilk (200 mL) European studies
2. Rapeseed oil (10 g); p95 analysis Germany +breastmilk (200 mL) European studies
3. Rapeseed oil (10 g); max. proposed level (EU) + breastmilk (200 mL) European studies

The median bodyweight of the WHO Growth Standards for 8 months old girls (7.9 kg) was used to calculate the total daily exposure per kg BW.

## 3 RESULTS

**FIGURE 2:**
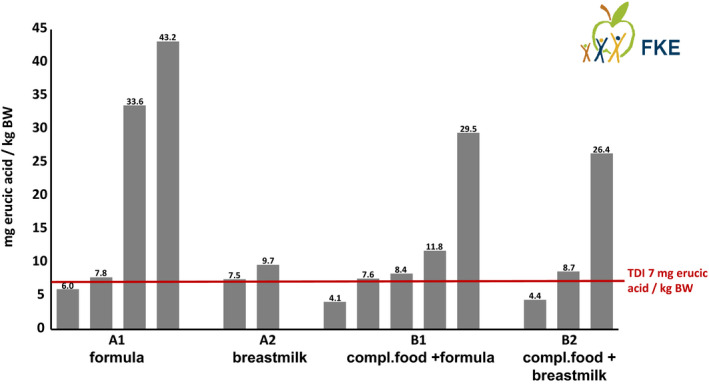
Overview of the results of 14 exposure scenarios in comparison with the TDI

The different available data sources allowed to calculate a total of 14 exposure scenarios. The results of the 6 scenarios for the first phase of exclusive milk feeding are shown in Table 2, and the results of the 8 scenarios for the second phase of complementary feeding +milk are shown in Table 3. An overview of the results compared with the TDI is shown in Figure 2 (Kalhoff et al., 2021).

**TABLE 2:** Scenarios for exclusive milk feeding (formula or breastmilk [BM])

			TDI: Erucic acid 7 mg/kg BW
A1: Formula	Selected data sources	Data values	Exposure: Erucic acid mg/kg BW
1	Consumption p50 × 12% powder in 100 g formula x MB concentration (EFSA samples infant formula powder)	0.199 kg/kg × 0.12 × 253 (mg/kg)	6.04 mg/kg
2	Consumption p95 × 12% powder in 100 g formula x MB concentration (EFSA samples infant formula powder)	0.257 kg/kg × 0.12 × 253 (mg/kg)	7.80 mg/kg
3	Consumption p50; EU maximum level for fat in 100 g formula (4,200 mg) x EU permitted maximum concentration of erucic acid in fat (0.4%)	0.199 kg/kg × (4,200 mg/0.1 kg) × 0.004	33.6 mg/kg
4	Consumption p95; EU maximum level for fat in 100 g formula (4,200 mg) x EU permitted maximum concentration of erucic acid in fat (0.4%)	0.257 kg/kg × (4,200 mg/0.1 kg) × 0.004	43.2 mg/kg
A2: BM			
1	Consumption p50 estimated (formula p50—10%); concentration (European studies)	0.179 kg/ /kg × (0.001 × 42,000 mg/kg)	7.52 mg/kg
2	Consumption p95 estimated (formula p95—10%); concentration (European studies)	0.231 kg/kg × (0.001 × 42,000 mg/kg)	9.70 mg/kg

**TABLE 3:** Scenarios for complementary feeding (CF) + milk (Formula, Breastmilk [BM])

	Selected data sources	Data values		TDI: Erucic acid 7 mg/kg BW
B1: CF+Formula		Rapeseed oil (p50: 2.67 g/kg) (p95: 6.06 g/kg) + formula	Exposure total	Exposure/kg BW
1	10 g rapeseed oil; p50 German samples; + follow‐on formula 200 mL (EU samples)	26.7 mg + 200 mL × 0.03 (mg/mL)	32.7	4.14
2	10 g rapeseed oil; p50 German samples:; + follow‐on formula 200 mL (EU permitted maximum)	26.7 mg + 200 mL × 0.167 (mg/mL)	60.1	7.61
3	10 g rapeseed oil; p95 German samples; + follow‐on formula 200 mL (EU samples)	60.1 mg + 200 mL × 0.03 (mg/mL)	66.1	8.37
4	10 g rapeseed oil; p95 German samples; + follow‐on formula 200 mL (EU permitted maximum)	60.1 mg + 200 mL × 0.167 (mg/mL)	93.5	11.8
5	10 g rapeseed oil (max. proposed level EU) + follow‐on formula 20 mL (EU permitted maximum)	200 mg + 200 mL × 0.167 (mg/mL)	233.4	29.5
B2: CF +BM				
1	10 g rapeseed oil; p50 German samples; + BM 200 mL (European studies)	26.7 mg + 200 mL × (0.001 × 42 mg/mL)	35.1	4.44
2	10 g rapeseed oil; p95 German samples +BM 200 mL (European studies)	60.1 mg + 200 mL × (0.001 × 42 mg/mL)	68.5	8.7
3	10 g rapeseed oil; proposed EU maximum; + BM 200 mL (European studies)	200 mg + 200 mL × (0.001 × 42 mg/mL)	208.4	26.4

Of the total 14 scenarios, only 3 scenarios resulted in exposures of erucic acid that were definitively below the EFSA TDI. The two by far lowest exposures (erucic acid around 4 mg/kg BW) were from the complementary feeding phase assuming the P50 value of the recent German rapeseed oil survey and breast milk or infant formula as marketed for the milk part (Table 3).

In contrast, for exclusive milk feeding around the end of the first month of life, the EFSA TDI was exceeded in almost all scenarios for both breastmilk and infant formula, especially assuming high consumption (P95) (Table 2).

Scenarios with the current EU maximum permitted concentration values for formula or vegetable oils uniformly resulted in multiples of the TDI, especially when combined with high consumption.

## 4 DISCUSSION

### 4.1 Overall evaluation

The present calculations are based on current European and German data on erucic acid concentrations in food and on precise consumption data as recommended in the German guidelines for infant feeding. The fact that only 3 of the 14 scenarios resulted in exposures significantly below the TDI for erucic acid raises doubts as to whether a nutritionally safe diet in infancy is sufficiently safe if toxicological considerations for erucic acid are taken into account. Since the Dietary Scheme has been the basis of nutritional counselling in Germany for decades, these concerns are of public health relevance.

While the data basis for the consumption part of the scenarios should be clear in the form of the dietary guidelines, for the concentration part, there is only clarity for erucic acid in rapeseed oil marketed in Germany. In contrast, the data on the concentration of erucic acid in breast milk and formula are not sufficiently available for a reliable assessment. Therefore, some of the present results are preliminary.

### 4.2 Rapeseed oil in infant nutrition

Rapeseed oil has nutritional and preventive benefits due to its unique fatty acid composition with a favorable ratio of omega‐3 and omega‐6 polyunsaturated fatty acids, a high oleic acid content, and a low proportion of saturated fatty acids. For this reason, rapeseed oil is recommended in Germany not only for infant nutrition, but also for children, adolescents, and adults, including pregnant and breastfeeding women (FKE, 2019a, 2019b; and DGE, 2019). In Germany, rapeseed oil is the most popular cooking oil in households with children (UFOP, 2017). Rapeseed oil is also the most frequent fat ingredient in complementary meals in Germany, whether in homemade meals or commercial products (Stimming et al., 2015).

Exclusive breastfeeding is considered the “gold standard” for infant nutrition in the first months of life. For nonbreastfed infants, EU food law on infant and follow‐on formula ensures safe nutrition. However, neither milk feeding regime is likely to guarantee safety with respect to erucic acid. This holds true even if only medium consumption and medium erucic acid contents were assumed in the scenarios. In both milk feeding options, the source of erucic acid is probably rapeseed oil. This oil may be one of the several fats present in formula to meet EU fatty acid content regulations, or erucic acid is transferred to breastmilk through maternal consumption of rapeseed oil.

Whether the data on erucic acid content for the milk part of the “Dietary Scheme” which we have taken from studies in European countries for breastmilk and from EFSA for formula (EFSA, 2016), actually reflect the current situation in Germany, we cannot say due to a lack of corresponding data. This is especially true for erucic acid levels in breastmilk because of the popularity of rapeseed oil in German households with children.

In the complementary feeding phase, rapeseed oil may become a direct source of erucic acid in infant nutrition. Based on the Dietary Scheme in Germany, this is the case for complementary meals irrespective of whether they are prepared at home or used as commercially produced baby food in jars. Fortunately, an up‐to‐date and representative analytical data base for erucic acid in rapeseed oil is available on the German retail market to estimate erucic acid exposure in complementary feeding (Russo et al., 2021). Remarkably, although the p95 erucic acid value of rapeseed oils in Germany is far below the just lowered EU maximum value for vegetable oils, the TDI is definitely exceeded when these oils are used in complementary meals that follow the recipes of the Dietary Scheme.

### 4.3 Erucic acid and the TDI

Erucic acid is a monounsaturated omega‐9 fatty acid, denoted 22:1ω9, with the chemical formula CH_3_(CH_2_)_7_CH=CH(CH_2_)_11_COOH. It is mainly prevalent in the seeds of Brassicaceae species, especially in rapeseed oil, and even more in mustard oil (Vetter et al., 2020; Wendlinger et al., 2014). Formerly, rapeseed oil was unsuitable for humans because of its high erucic acid content (HEAR). Due to breeding successes, low‐erucic acid rapeseed (LEAR) oils have been considered safe for human consumption since the 1970s.

European Food Safety Authority's assessment of health risks from exposure to erucic acid from food is based on animal models with the heart as the target organ and myocardial lipidosis as the most sensitive endpoint (EFSA, 2016). Lipidoses triggered by erucic acid in animals are reversible; they have not been observed in humans. From the highest dose of chronic erucic acid exposure at which no adverse effects were observed (NOAEL), that is, 0.7 g erucic acid/kg BW, and a safety factor of 100 for transfer to humans, EFSA determined a TDI of 7 mg/kg BW and day. In principle, our results confirm EFSA's exposure assessment with the potentially highest exposure to erucic acid in early childhood.

## 5 CONCLUSION

The TDI is set assuming chronic exposure throughout life. In the course of human development, the energy demand and thus the amounts of food per kg BW are highest in early childhood, a particularly sensitive developmental phase of infancy. Our results are of concern because the high exposure to erucic acid calculated in the scenarios occurs daily for many months during this sensitive phase.

The calculations presented show that the nutritionally safe guidelines for infant feeding in Germany do not seem to offer such safety from a toxicological point of view with regard to erucic acid, if the TDI is used as an absolute benchmark for evaluation. Since we have used the just drastically lowered EU maximum values of erucic acid in vegetable oils and formula for some of the scenarios, there is further need for clarification.

Therefore, the question arises to what extent the documented low‐erucic acid concentration in the rapeseed oil supply in Germany is also reflected in low‐erucic acid concentrations in breast milk and infant formula. Until these analyses are available, it is not possible to definitely quantify the potential risk from exposure to erucic acid during infancy and to weigh it against the documented safe nutritional profiles of the standard infant nutrition guidelines and the use of rapeseed oil in Germany.

## ETHICAL APPROVAL

The authors declare that they do not have any conflict of interest. This study does not involve any human or animal testing.

## Data Availability

The data that support the findings of this study are available from the corresponding author upon reasonable request.
